# Hydrophobically Coated Superparamagnetic Iron Oxides Nanoparticles Incorporated into Polymer-Based Nanocapsules Dispersed in Water

**DOI:** 10.3390/ma13051219

**Published:** 2020-03-09

**Authors:** Elżbieta Gumieniczek-Chłopek, Joanna Odrobińska, Tomasz Strączek, Agnieszka Radziszewska, Szczepan Zapotoczny, Czesław Kapusta

**Affiliations:** 1 Faculty of Physics and Applied Computer Science, AGH University of Science and Technology, Mickiewicza Av. 30, 30-059 Krakow, Poland; echlopek@agh.edu.pl (E.G.-C.); t.straczek@gmail.com (T.S.); 2 Faculty of Chemistry, Jagiellonian University, Gronostajowa 2, 30-387 Krakow, Poland; odrobinska@chemia.uj.edu.pl; 3 Faculty of Metals Engineering and Industrial Computer Science, AGH University of Science and Technology, Mickiewicza Av. 30, 30-059 Krakow, Poland; radzisze@agh.edu.pl

**Keywords:** polymer nanocapsules, superparamagnetic nanoparticles, core–shell nanoparticles, chitosan derivative

## Abstract

This paper reports the characterization of iron oxide magnetic nanoparticles obtained via the thermal decomposition of an organometallic precursor, which were then loaded into nanocapsules prepared via the emulsification process in the presence of an amphiphilic derivative of chitosan. The applied synthetic method led to the formation of a hydrophobic layer on the surface of nanoparticles that enabled their loading in the hydrophobic liquid inside of the polymer-based capsules. The average diameter of nanoparticles was determined to be equal to 15 nm, and they were thoroughly characterized using X-ray diffraction (XRD), magnetometry, and Mössbauer spectroscopy. A core–shell structure consisting of a wüstite core and maghemite-like shell was revealed, resulting in an exchange bias effect and a considerable magnetocrystalline anisotropy at low temperatures and a superparamagnetic behavior at room temperature. Importantly, superparamagnetic behavior was observed for the aqueous dispersion of the nanocapsules loaded with the superparamagnetic nanoparticles, and the dispersion was shown to be very stable (at least 48 weeks). The results were analyzed and discussed with respect to the potential future applications of these nanoparticles and nanocapsules based on biopolymers as platforms designed for the magnetically navigated transport of encapsulated hydrophobic substances.

## 1. Introduction

Combining medical methods with materials and tools of nanotechnology led to the formation of the dynamically developing field of science known as nanomedicine. In 1960, Freeman et al. presented the pioneering idea of the application of magnetic materials and magnetic fields in medicine [1]. Such approaches, in solving some biomedical problems in diagnostics, treatment, and regeneration, can significantly improve the curing of patients [2]. In particular, there is increasing interest in the application of magnetic nanoparticles (MNPs). In in vivo applications, the created magnetic systems can play a therapeutic or diagnostic role (in controlled drug delivery, as hyperthermia treatment, or as contrast agents in nuclear magnetic resonance imaging, MRI), while in vitro applications include magnetic bioseparation, as well as the labeling and detection of various biomolecules [3,4,5,6,7,8,9,10,11]. For the biomedical applications, the most common and safe magnetic nanoparticles are based on iron oxides. Their limited toxicity, together with commonly applied biocompatible coatings, allows meeting the respective requirements concerning the adequate dose of particles. Furthermore, biomedical applications require high magnetization values and sizes typically smaller than 100 nm with a narrow distribution, which is possible to achieve with iron oxide nanoparticles [12,13,14,15,16]. An important aspect is also the use of an appropriate biocompatible coating; hydrophilic coatings are commonly based on polysaccharides and polypeptides, while hydrophobic coatings are commonly based on surfactants [16,17,18]. Particle size reduction may affect the ratio between the thermal energy and the product of magnetic anisotropy and particle volume, such that the superparamagnetic limit can be crossed, which strongly determines the magnetic behavior of the particle. Below a certain temperature, termed the blocking temperature, magnetic moments are frozen, such as in a ferro- or ferrimagnet; above this temperature, the resultant moments of individual nanoparticles fluctuate between easy magnetization directions. The effect is similar to that of atomic moments in a paramagnet, but it corresponds to 1000-fold bigger magnetic moments [19]. Moreover, by decreasing the size of nanoparticles, the percentage of surface atoms increases, which gives rise to the second key issue related to the magnetic properties of nanoparticles, i.e., surface effects [20]. A high surface-to-volume ratio induces a tendency to agglomeration and renders nanoparticles highly sensitive to oxidizing agents, resulting in them reacting easily with chemicals. Adequate selection of the synthesis method, as well as surface coating, may limit aggregation, promote dispersibility in water, and enable further functionalization of the particles, which is vital for their biomedical application [21,22,23]. Hydrophobic coatings enable the dispersibility of particles in various nonpolar solvents and limit their aggregation, which leads to better monodispersity of their sizes, thereby allowing controlled properties [24,25,26]. To obtain such features, carboxylic acids or alkyl phosphates are most often used, while oleic acid was shown to offer very good stability and dispersibility of coated nanoparticles in nonpolar solvents [27]. These size restrictions can be overcome in the high-temperature thermal decomposition method. By using organic solvents boiling at high temperatures, MNPs of narrow size distribution and high crystallinity can be obtained, and the nanoparticles synthesized can consist of different iron oxide compounds. In both types of synthesis method, environmental factors such as the pH, temperature, presence of an inert gas, and reaction time are extremely important [4].

Incorporation of MNPs inside biocompatible and/or biodegradable systems designed for controlled delivery can significantly improve some treatment or diagnostic methods thanks to the possibility of navigating systems using a magnetic field. Targeted drug delivery through blood vessels mainly occurs via the hydrodynamic forces of blood flow, whereas systems with magnetic properties can additionally be supported remotely using an external magnetic field. Ultimate systems could be magnetically transported to the targeted tissue, as well as immobilized magnetically while the drug is released at the site [13,28]. By applying a magnetic field, such carriers are able to achieve the desired position in a more precise and controllable way [29,30]. Furthermore, such platforms can also be used for magnetically controlled (bio)reactors, enabling reactions between components transported via facile magnetic remoting, even with nanoliter volumes [21,22,23,24,25,26,27,28,29,30,31,32,33,34]. We only recently presented polymer-based nanocapsules stabilized using a modified polysaccharide with an oleic acid liquid containing suspended MNPs, showing that they can be navigated using a static magnetic field [34]. Here, we present detailed studies on similar nanocarriers but with novel MNPs obtained via the thermal decomposition method, with a dispersion in oleic acid formed at a very high concentration that was previously inaccessible. Importantly, we also show that the applied synthetic method leads to the formation of nanoparticles with a core–shell structure and hydrophobic coating, which are crucial elements for the efficient encapsulation and tunable magnetic properties of the capsules. Furthermore, the obtained magnetic capsules in an aqueous dispersion were shown to be very stable, which is very important for their future potential application in the fabrication of smart magnetically driven delivery systems.

## 2. Materials and Methods

### 2.1. Materials

Chitosan (molecular weight 50–190 kDa, based on viscosity measurements), glycidyltrimethylammonium chloride (GTMAC, technical, ≥90%), *n*-dodecyl aldehyde (92%), and sodium cyanoborohydride (95%) were all from Sigma Aldrich and used as received. Anhydrous iron(III) chloride (Merck, anhydrous for synthesis), acetic acid (Chempur, 99.5%), sodium oleate (TCI, >97.0%), 1-octadecene (Alfa Aesar, technical, 90%), oleic acid (OA, Alfa Aesar, technical, 90%), and *n*-octadecane (p.a., Polyscience Corp.) were also used as received. During the synthesis, ethanol (96%), acetone (analytical grade), hexane (analytical grade), and methanol (analytical grade), all from Chempur, and deionized water were used. Cellulose tubing with a 14,000 g/mol cut-off (Sigma Aldrich, St. Louis, MI, USA) was used for dialysis.

#### 2.1.1. Synthesis of Chitosan Derivative (CChit-C12)

Synthesis of *N*-[(2-hydroxy-3-trimethylamine) propyl] chitosan chloride and further dodecyl group modification were carried out using the modified procedure described by Karewicz et al. [35]. Briefly, 17 mL of glycidyltrimethylammonium chloride was added to 160 mL of a 2% (*w*/*v*) solution of chitosan dissolved in 1% acetic acid. The reaction mixture was kept at 70 °C for 48 h. The resulting solution was purified by precipitation into a 50:50 (*v*/*v*) cooled mixture of acetone and methanol. The precipitate was centrifuged and washed three times with acetone and then three times with ethanol. To remove unreacted residues, the precipitate was dissolved in deionized water and centrifuged. The received supernatant was precipitated with a mixture of acetone and ethanol (4:1, *v*/*v*). Then the precipitate was washed with ethanol, centrifuged, and dissolved in deionized water. The obtained mixture was dialyzed in water for four days. The resulting derivative was freeze-dried. The degree of substitution by quaternary ammonium groups was determined using conductometric titration with 0.017 M AgNO_3_. At the second stage, 1 g of the previously modified chitosan was dissolved in a 1:1 mixture of methanol and a 1% aqueous solution of acetic acid. Then, 0.3 mmol of *N*-dodecyl aldehyde and 20 mmol of sodium cyanoborohydride were dissolved in 20 mL of methanol and added to the chitosan solution. The reaction mixture was stirred at 20 °C until the sol formation was observed (36 h). The resultant suspension was precipitated by adding a methanol/diethyl ether (50:50 *v*/*v*) mixture. The white precipitate was washed several times with methanol and subsequently with diethyl ether. Afterward, the product was dissolved in deionized water and dialyzed for four days in a mixture of water and methanol (*v*/*v* 3:1) and for the next four days in water. The resultant amphiphilic derivative of chitosan was freeze-dried. Determination of the degree of substitution of *N*-dodecyl groups was based on the intensity ratio of the ^1^H-NMR signals of the five ring-skeleton protons in the range of 3.4–4.4 ppm and 10 alkyl chain protons in the region of 1.15–1.25 ppm.

#### 2.1.2. Synthesis of Iron(III) Oleate Complex

The metal oleate complex was prepared according to the procedure described by Leszczyński et al. [36], mixing 30 mL of deionized water, 40 mL of ethanol 96%, 70 mL of hexane 96%, 3.25 g of anhydrous iron(III) chloride, and 18.25 g of sodium oleate. The resulting solution was heated to 60 °C and kept at that temperature for 5 h. The dark hydrophobic phase was separated in a separator funnel, washed with deionized water, and heated to 40 °C to evaporate hexane.

#### 2.1.3. Synthesis of Iron Oxide Nanoparticles

Firstly, 42 mL of 1-octadecene, 1.1 mL of oleic acid, and 7 g of iron(III) oleate were poured into a three-neck flask. The reaction was carried out as described by Park et al. [37] in an anaerobic atmosphere with incessant stirring and increasing temperature. When the mixture reached 318 °C, it was kept at this temperature for 45 min. The resulting solution was cooled to room temperature, washed several times with ethanol, and separated from the unreacted residues by sonication in hexane and centrifugation. Finally, the obtained nanoparticles were dried under vacuum.

#### 2.1.4. Preparation of Capsules with Magnetic Nanoparticles Suspended in Oleic Interior

Preparation of the capsules, based on the emulsification process with self-assembly of the amphiphilic chitosan derivate on the dispersed oil nanodroplets, followed the method previously described by us [38,39] with some modifications. Briefly, 10 μL of oleic acid with dispersed magnetic nanoparticles (ca. 100 g/L) was added to the solution of the chitosan derivative, CChit-C12, in 0.15 M NaCl (1 g/L). The emulsification was realized using a vortex shaker and 30 min of pulse ultrasonication (1 s on and 2 s off). Non-encapsulated magnetic nanoparticles were removed by magnetic separation. To obtain a more concentrated suspension of the capsules with a stronger magnetic response required for the magnetometry measurements, a similar procedure but with different concentrations and amounts of components was done. In this case, 30 μL of oleic acid with dispersed MNPs (100 g/L) was added to 1 mL of chitosan derivative, CChit-C12, in 0.15 M NaCl (10 g/L).

#### 2.1.5. Preparation of Capsules for Scanning Transmission Electron Microscopy (STEM) Imaging

For scanning transmission microscope imaging, capsules were prepared similarly to the method previously presented by us [34], except that 7.5 μL of *n*-octadecane with dispersed magnetic nanoparticles (50 g/L) was added, and the sonication process was carried out in the 30–33 °C temperature range.

### 2.2. Methods

#### 2.2.1. Hydrodynamic Diameter and Zeta Potential Measurements

A Zetasizer Nano ZS instrument (Malvern Instruments, Worcestershire, UK) working at a 173° detection angle was used for dynamic light scattering (DLS) measurements of nanoparticles (dispersed in hexane) and capsules (dispersed in water). All measurements were performed at 22 °C, and the reported data represent the averages of three series of measurements (10–100 runs each) and their standard deviations. Zeta potentials of the capsules were also determined with the Malvern Zetasizer Nano ZS apparatus using laser doppler velocimetry (LDV).

#### 2.2.2. Scanning Transmission Electron Microscopy

A Nova NanoSEM 450 (FEI, Eindhoven, the Netherlands) scanning electron microscope (SEM) operating at 30 keV was used for imaging the nanoparticles and the capsules. The observations were carried out using scanning transmission electron microscopy mode (STEM). The estimated average diameter was calculated using the program Fiji-ImageJ.

#### 2.2.3. X-Ray Diffraction Measurements

The crystal structure of the prepared nanoparticles and their crystallite size were characterized at room temperature with Cu Kα radiation using a Siemens D5000 X-ray diffractometer (Siemens, Munich, Germany) equipped with a graphite monochromator.

#### 2.2.4. Vibrating Sample Magnetometry Measurements

Magnetic properties of vacuum-dried nanoparticles and capsules were measured using a vibrating sample magnetometer of the Quantum Design Physical Property Measurement System. Magnetization measurements were performed in an applied magnetic field in the 4–300 K temperature range. The field-cooled (FC) and zero-field-cooled (ZFC) susceptibility curves were taken in this temperature range at a 100 Oe magnetic field strength.

#### 2.2.5. Mössbauer Spectroscopy

^57^Fe Mössbauer measurements were carried out in transmission mode at constant acceleration in a spectrometer. A 50-mCi ^57^Co/Rh source was used.

#### 2.2.6. Other Methods

Characterization of chitosan derivative via conductometric titration was performed with a multifunctional measuring apparatus CPC–505 (Conbest, Krakow, Poland) using a platinum indicator electrode. ^1^H-NMR spectra were recorded on a Bruker Avance III HD 400 MHz in D_2_O with CD_3_COOD. Fourier-transform infrared (FTIR) spectra were recorded using an ALPHA FTIR spectrometer (Bruker, Billerica, MA, United States) working in attenuated total reflectance (ATR) mode (on diamond). For thermal analyses, a STA 449F3 instrument (Netzsch, Selb, Germany) was used.

## 3. Results and Discussion

### 3.1. Characterization of Chitosan Derivative

**Figure 1:**
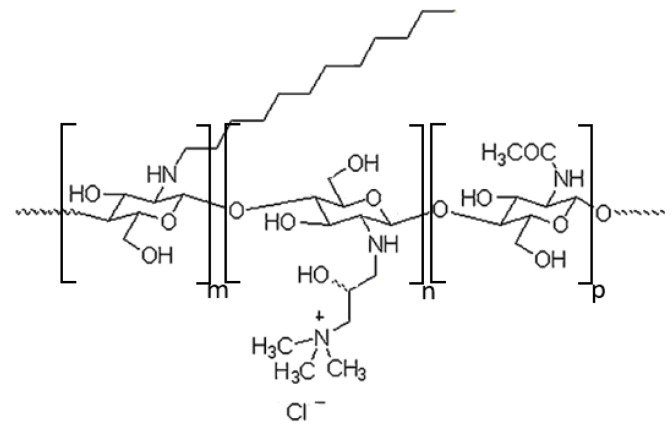
Scheme of chitosan derivative (CChit-C12) containing quaternary ammonium and *N*-dodecyl groups.

The amphiphilic chitosan derivative containing quaternary ammonium and *N*-dodecyl groups (Figure 1) was synthesized and used later to stabilize the oil nanodroplets. The degree of substitution by quaternary ammonium groups was determined to be 63% ± 2% [40]. The determination of degree of substitution of *N*-dodecyl groups based on the ^1^H-NMR signals provided a value of 3% (Figure S1, Supplementary Materials) [35]. The assignment of other signals of CChit-C12, as well as some residual solvent peaks in the ^1^H-NMR spectrum, is presented in Figure S1 (Supplementary Materials).

### 3.2. Characterization of Magnetic Nanoparticles

**Figure 2:**
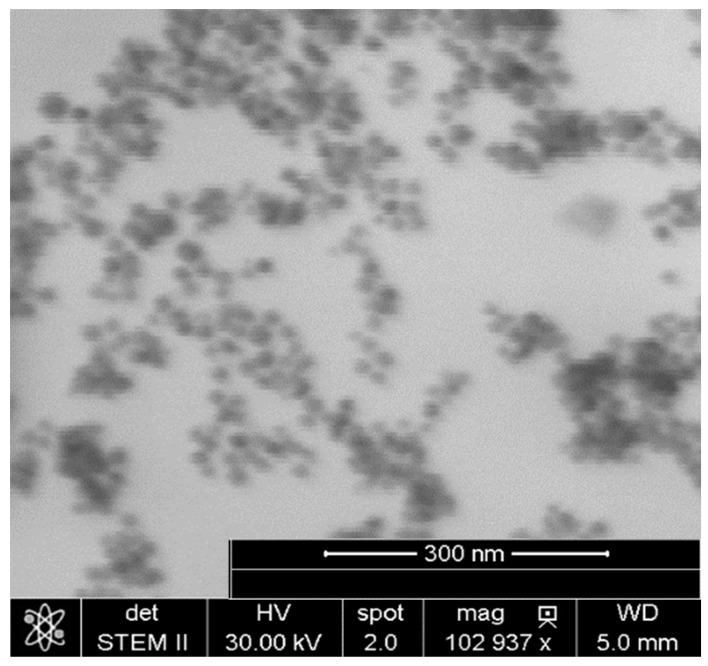
Scanning transmission electron microscopy (STEM) image of the magnetic nanoparticles.

The obtained MNPs were dried and imaged by STEM (Figure 2). Their average diameter was estimated to be equal to 15 nm based on the images. The applied synthetic method implied the formation of a hydrophobic oleic acid coating on their surface. To confirm the presence of oleic acid, FTIR measurements were performed (Figure S2, Supplementary Materials). In the spectra of both pure oleic acid and the nanoparticles coated with it, four sharp bands could be observed. For pure oleic acid, the peaks at 2922 and 2853 cm^−1^ were attributed to asymmetric CH_2_ stretching and symmetric CH_2_ stretching, whereas the intense peak at 1710 cm^−1^ was assigned to stretching of the C=O bond. The weak signal at 3000 cm^−1^ was assigned to C–H stretching in the C=C–H group. For nanoparticles coated with oleic acid, the locations of characteristic bands were shifted to a lower frequency range. The peak characteristic of C=O in the carboxyl group was absent, and two new bands at 1560 and 1645 cm^−1^ appeared, corresponding to asymmetric and symmetric stretching in –COO^–^ groups. The difference in wavenumber between those bands indicated the chelating bidentate, where the –COO^–^ groups were linked with Fe ions. The thermogravimetry analysis (TGA)/differential scanning calorimetry (DSC) measurements carried out at temperatures up to 1000 °C (Figure S3, Supplementary Materials) showed a mass loss of 8% occurring mostly up to 400 °C, with a maximum rate close to 260 °C, accompanied by an exothermic peak corresponding to the oleic acid desorption process [41,42,43].

The X-ray diffraction pattern of the nanoparticles (Figure S4, Supplementary Materials) revealed the presence of magnetite and wüstite phases. The lines corresponding to the magnetite phase (at 2θ ≈ 30° and 77°) showed considerable width, *W*(2θ) ≈ 2°, with no measurable line shift with respect to the diffractogram of microcrystalline magnetite. The crystallite size *d* evaluated from this linewidth using Scherrer’s equation, *d* ≅ *K*λ/(*W*(2θ)cos(θ)), amounted to 4.4 nm. The approximate value of Scherrer’s constant K of 1 and the wavelength λ of the Cu *K*α radiation of 0.154 nm were taken. A smaller linewidth of 1.5° observed for wüstite and its crystallite size of 6 nm, slightly bigger than for magnetite, were derived [44]. A slight shift of the lines corresponding to the wüstite phase toward larger angles indicated a smaller lattice constant, which could be interpreted as originating from compressive stress in a possible core–shell-like structure with wüstite in the core and magnetite in the shell. In such a structure, the maghemite shell could be formed as a result of oxidation of the wüstite phase, which is supported by the fact that wüstite is a metastable phase which does not exist in stoichiometric form in ambient conditions. FeO shows a strong tendency toward a disproportionation reaction to iron and magnetite or oxidation to magnetite or maghemite. Regarding the obtained FeO crystallite size of 6 nm as the core size and the magnetite/maghemite crystallite size of 4.4 nm as the shell thickness, we obtained an average particle diameter of 14.8 nm, very close to that determined from STEM images. The stress at the phase boundary, deduced from the FeO diffraction line shift, may lead to increased magnetocrystalline anisotropy [45,46,47,48,49,50].

**Figure 3:**
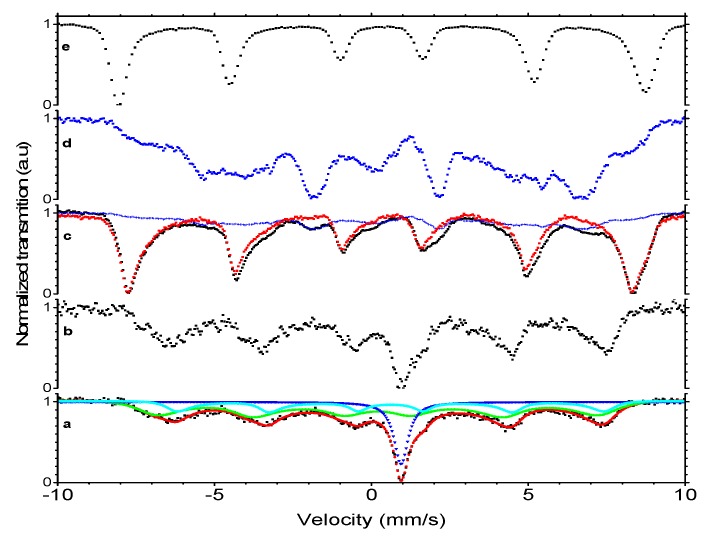
Mössbauer spectra of the magnetic nanoparticles (experimental points are represented by black dots): (**a**) at room temperature (RT) with the fit of wüstite singlet (blue), magnetite sextets (light blue and green), and their sum (red); (**b**) at RT after eight months of sample storage; (**c**) at liquid nitrogen temperature (LNT) with wüstite contribution (blue) and the spectrum after its subtraction (red); (**d**) the spectrum of microcrystalline wüstite at LNT; (**e**) the spectrum of microcrystalline maghemite, γ-Fe_2_O_3_, at LNT.

The Mössbauer spectra (Figure 3) were obtained for the dried nanoparticle material at room temperature and at liquid nitrogen temperature (77 K).

The spectrum obtained at room temperature consisted of a broad, structured magnetic sextet and a singlet, which could be attributed to the magnetite and wüstite phases, respectively. The characteristic broadening of the lines toward the center indicated a relaxational character of the spectrum due to magnetic fluctuations faster than the lifetime of the Mössbauer excited level. Fitting of the spectrum with a singlet and two relaxational sextets according to the model proposed by Blume and Tjon [51] provided the parameters (Table 1) corresponding to Fe^2+^ in wüstite (central singlet, 21% intensity) and Fe^3+^ at the tetrahedral and octahedral positions in magnetite (dominant relaxational sextet, 55% intensity), as well as Fe^2.5+^ at octahedral positions in magnetite (minor relaxational sextet, 23% intensity). The intensity ratio of the Fe^3+^ to Fe^2.5+^ components of 2.4, nearly five times higher than 0.5 observed for stoichiometric magnetite, revealed a considerable oxidation of the magnetite shell of nanoparticles toward maghemite, γ-Fe_2_O_3_, i.e., magnetite with octahedral Fe vacancies in its inverse spinel crystal structure.

In the spectrum obtained at liquid nitrogen temperature, the lines were much narrower, indicating its static character. The wüstite contribution was no longer a singlet, but represented a structured, magnetically split pattern. After the subtraction of the microcrystalline wüstite spectrum in the amount of 22% of the total integral intensity (as determined from the room temperature fit) from the spectrum of nanoparticles, the remaining part resembled the spectrum of microcrystalline maghemite (Figure 3).

The fluctuation frequency of 52 MHz obtained from relaxational fits was rather low and correlated well with the relatively high blocking temperature of 275 K obtained from the FC/ZFC magnetic susceptibility measurements. A relatively high asymmetry parameter, ρ = 0.95, which reflects the relative residence time of the nanoparticle moment along the easy magnetization direction, indicated a high magnetocrystalline anisotropy of these nanoparticles [49,52,53,54,55].

**Figure 4:**
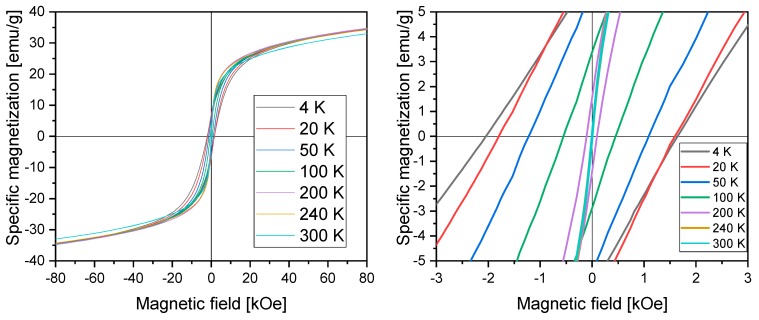
Magnetization vs. magnetic field in the 4–300 K temperature range of magnetic nanoparticles (MNPs).

Figure 4 presents results of magnetization measurements performed in an applied magnetic field in the 4–300 K temperature range.

The results showed no saturation up to 80 kOe and hysteresis loops at the origin, with the values of coercivity and remanence decreasing with increasing temperature (up to the range of the Néel temperature of FeO) (Table 2). The shapes of curves at 240 and 300 K showed vanishing hysteresis, which indicated the superparamagnetic properties of the material at these temperatures and higher [19]. Magnetization did not reach saturation in the highest field of 80 kOe and a lack of saturation was observed even above the Néel temperature of FeO, indicating a strong magnetocrystalline anisotropy of the maghemite shells, consistent with Mössbauer results. This can be attributed to a considerable number of octahedral vacancies in the maghemite shell and/or to the effect of the magnetically inert oleic acid coating, which not only ensured environmental protection but also brought about surface effects. The presence of the OA shells resulted in a decrease in the effective magnetization of nanoparticles in comparison with the bulk value, which could contribute to an enhancement of the magnetic anisotropy. Considering the interaction between the antiferromagnetic (AFM) FeO core and ferrimagnetic (FIM) maghemite shell of the magnetic nanoparticles, which is strongly related to the core size and shell thickness, the presence of exchange coupling was expected. Large values of coercivity below the Néel temperature and a shift of hysteresis loops toward negative field values indicated the presence of exchange bias coupling across the AFM core/FIM shell interface [45,46,56]. Both the exchange bias effect and the coercivity enhancement below the Néel temperature make these nanoparticle materials very interesting magnetic system.

Virgin magnetization curves in the 4–300 K temperature range (Figure S5, Supplementary Materials) showed a significant increase in magnetization at low magnetic field values, which indicated the nucleation type of magnetization reversal mechanism. The nucleation process can be observed in materials with a low number of defects [57,58,59].

**Figure 5:**
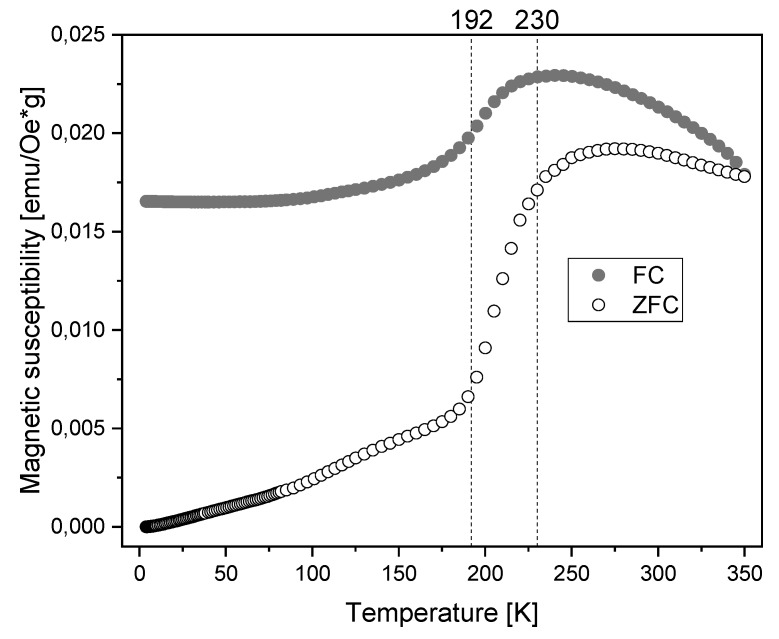
Magnetic susceptibility of MNPs as a function of temperature under 50 Oe magnetic field.

Figure 5 shows the zero-field-cooled (ZFC) and field-cooled (FC) magnetic susceptibility curves.

In the antiferromagnetic state of the FeO core, for temperatures below the Néel temperature (T_N_ ≈ 192 K), the susceptibility showed small values, whereas, at 230 K, a significant increase occurred. The shift in Néel temperature to a value higher than reported for bulk FeO is related to the presence of the inverse spinel crystal structure as the nanoparticle shell, which is magnetically ordered at this temperature [50]. The maximum of the ZFC curve can be regarded as corresponding to the blocking temperature. Below the blocking temperature, magnetic moments are frozen, and particles do not exhibit superparamagnetic properties. Above the blocking temperature, magnetic moments of the nanoparticles fluctuate with characteristic fluctuation time due to the ability to overcome the magnetocrystalline energy barrier [45,60]. The average blocking temperature of the investigated magnetic nanoparticles determined as the maximum of the ZFC curve was equal to 275 K. This is consistent with the results of magnetization measurements (hysteresis loops), where a vanishing hysteresis was observed at 300 K.

The presented results indicate that appropriate modifications of the synthesis conditions or post-synthesis treatment, the relative proportion of the wüstite core to maghemite shell, and the implied magnetic properties and dynamics of the nanoparticles can be controlled to adjust them to the requirements of diagnostic or therapeutic procedures.

### 3.3. Capsules with Magnetic Properties

Polymer-based capsules were prepared using cationically and hydrophobically modified chitosan derivatives that can anchor the oleic acid droplets, stabilizing the capsules without the use of low-molecular-weight surfactants that are commonly undesirable in biomedical applications [33]. For the preparation of the capsules, dispersion of the obtained magnetic nanoparticles in oleic acid (or in *n*-ocatadecane for STEM imaging) with a very high concentration (100 g/L) was used. Reaching such a high concentration in the hydrophobic interior of the capsules was crucial for their magnetic response and was achieved here due to the applied synthetic procedure.

**Figure 6:**
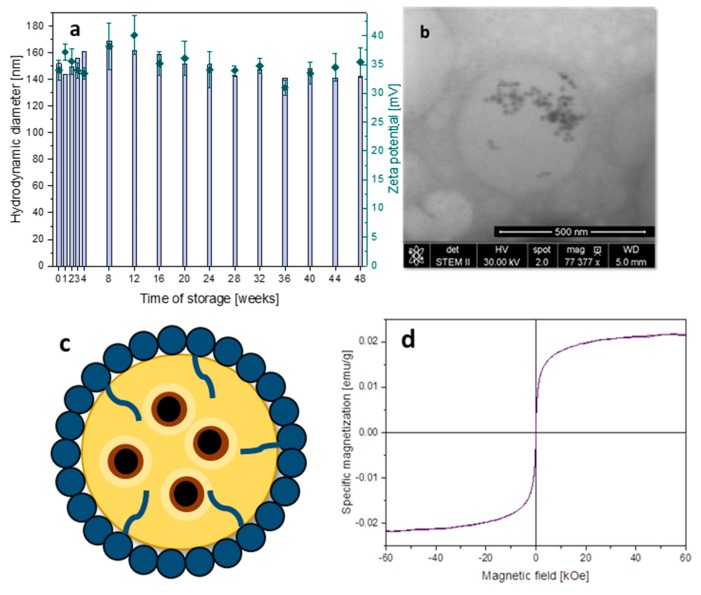
(**a**) Hydrodynamic diameter (columns, left scale) and potential zeta (points, right scale) of capsules with encapsulated MNPs over 48 weeks of storage; (**b**) STEM image of the capsule template on *n*-octadecane core with encapsulated MNPs; (**c**) scheme of the capsule with chitosan-based shells and magnetic nanoparticles embedded in the oil interior; (**d**) magnetization vs. magnetic field at 300 K of the aqueous dispersion of the capsules with encapsulated MNPs.

Figure 6a presents the values of hydrodynamic diameters and zeta potentials of the capsules with encapsulated magnetic nanoparticles over 48 weeks of storage at 4 °C. The obtained results indicated very good stability of the aqueous dispersion of the nanocapsules as determined by the high potential zeta values (above 33 mV). Importantly, their hydrodynamic diameters below 150 nm and size dispersity (polydispersity index (PDI) < 0.3) stayed almost unchanged for such a long storage time. Figure 6b confirms the structure of a spherical capsule able to encapsulate MNPs, while some aggregation of the nanoparticles observed here may have been the result of crystallization of the *n*-octadecane matrix after cooling it to room temperature. Figure 6c schematically presents the spherical capsule with magnetic nanoparticles encapsulated in its liquid interior. Figure 6d shows the magnetization vs. magnetic field at 300 K of concentrated capsules. The shape of the curve confirms the magnetic characteristic of the systems. Moreover, a lack of hysteresis indicates the superparamagnetic properties of the nanocapsules. Presence of the strong and well-defined magnetic properties of the designed carriers is a key aspect in the context of potential biomedical applications such as magnetically navigated novel drug delivery systems or MRI contrast.

## 4. Conclusions

Modified chitosan-based nanocapsules with a liquid oleic acid interior loaded with magnetic nanoparticles were successfully prepared and studied. The XRD, magnetometry, and Mössbauer spectroscopic studies revealed that the nanoparticles synthesized via the thermal decomposition method consisted of wüstite cores (ca. 6 nm in diameter) and maghemite shells (ca. 4.4 thick). Such a structure exhibits considerable coercivity and magnetocrystalline anisotropy with an exchange bias effect at temperatures below the Néel temperature of wüstite (ca. 192 K), while, at higher temperatures, relevant to their loading in dispersed nanocapsule, the nanoparticles show a superparamagnetic behavior. The obtained relatively long mean time of residence of nanoparticle moment at the easy direction of magnetization confirms a large magnetocrystalline anisotropy. A relatively strong magnetic response was observed for the nanocapsules loaded with the nanoparticles, with vanishing coercivity, indicating that the superparamagnetic properties of the nanoparticles are preserved. This was achieved even for a very high concentration of dispersed MNPs in oleic acid (ca. 100 g/L) that brought also relatively high magnetization of the aqueous dispersion of the capsules, which is necessary for their efficient magnetically driven navigation. The nanoparticles and nanocapsules presented here exhibit great potential for tailoring their magnetic properties and dynamics with respect to applications such as smart delivery carriers thanks to the tunable core–shell structure of the MNPs and the formation of a highly concentrated dispersion of nanoparticles in the liquid interior of the capsules [47]. Such systems, thanks to the application of biocompatible components, can be tested in biological environments for magnetically controlled delivery of active hydrophobic substances.

## Figures and Tables

**Table 1:** Hyperfine parameters obtained from the fits of Mössbauer spectra together with those for reference materials taken from the literature. **ρ** is the asymmetry parameter, i.e., the relative residence time of nanoparticle moment along its easy direction.

Type of Measurement	Isomer Shift * (mm/s)	Hyperfine Field (T)	χ^2^	Fluctuation Frequency (MHz)	ρ	Intensity (%)
**Magnetic nanoparticles**						
Room temperature	0.21(1)	44.7(2)	1.1	52(4)	0.95(1)	55(3)
	0.73(1)	43.2(2)				23(1)
	1.05(1)	0				22(2)
Liquid nitrogen temperature	0.30(3)	49.5(1)	0.22	assumed static	-	61(1)
	0.84(2)	48.6(3)				17(3)
	0.99(2)	-				22(2)
**Maghemite [38]**						
Room temperature	0.23	50				
	0.35	50				
Liquid helium temperature	0.4	52				
	0.48	53				
**Wüstite [38]**						
Room temperature	0.95					
	0.9					

* Relative to α-iron.

**Table 2:** Coercivity and remanence values determined from magnetization vs. magnetic field curves at different temperatures. The two coercivity values given correspond to the hysteresis loop intersection with a magnetic field axis at the negative and positive sides.

Temperature	Coercivity (kOe)	Remanence (emu/g∙cm^3^)
4 K	−2.05	6.4
	1.64	
20 K	−1.80	7.1
	1.59	
50 K	−1.22	5.7
	1.08	
100 K	−0.55	3.1
	0.45	
200 K	−0.11	1.5
	0.09	
240 K	−0.01	0.2
	0.01	
300 K	−0.00	0
	0	

## Supplementary Materials

The following are available online at https://www.mdpi.com/1996-1944/13/5/1219/s1: Figure S1. ^1^H-NMR spectrum of amphiphilic chitosan derivative containing quaternary ammonium and *N*-dodecyl groups; Figure S2. FTIR spectra of oleic acid and iron oxide nanoparticles coated with oleic acid; Figure S3. Thermal analysis (TGA/DSC) of nanoparticles coated with oleic acid; Figure S4. XRD diffraction patterns of magnetic nanoparticles, maghemite, and wüstite; Figure S5. Virgin magnetization curves in the 4–300 K temperature range.
