# Supplementary material for: Hydrophobically Coated Superparamagnetic Iron Oxides Nanoparticles Incorporated into Polymer-Based Nanocapsules Dispersed in Water
Source: Materials (Basel). 2020 Mar 9;13(5):1219. doi: 10.3390/ma13051219 (PMC7085046; doi:10.3390/ma13051219)
Supplement: Supplementary file 1 [file materials-13-01219-s001.pdf]

materials

Supplementary Information

Hydrophobically Coated Superparamagnetic Iron Oxides Nanoparticles Incorporated into Polymer-Based Nanocapsules Dispersed in Water

Elżbieta Gumieniczek-Chłopek [1,2], Joanna Odrobińska [2], Tomasz Strączek [1], Agnieszka Radziszewska [3], Szczepan Zapotoczny [2,*] and Czesław Kapusta [1,*]

[1] Faculty of Physics and Applied Computer Science, AGH University of Science and Technology, Mickiewicza Av. 30, 30-059 Krakow, Poland; echlopek@agh.edu.pl (E.G.-C.); t.straczek@gmail.com (T.S.);
[2] Jagiellonian University, Faculty of Chemistry, Gronostajowa 2, 30-387 Krakow, Poland; odrobinska@chemia.uj.edu.pl
[3] AGH University of Science and Technology, Faculty of Metals Engineering and Industrial Computer Science, Mickiewicza Av. 30, 30-059 Krakow, Poland; radzisze@agh.edu.pl
* Correspondence: zapotocz@chemia.uj.edu.pl (S.Z.), kapusta@agh.edu.pl (C.K.)

Received: 7 February 2020; Accepted: 2 March 2020; Published: date

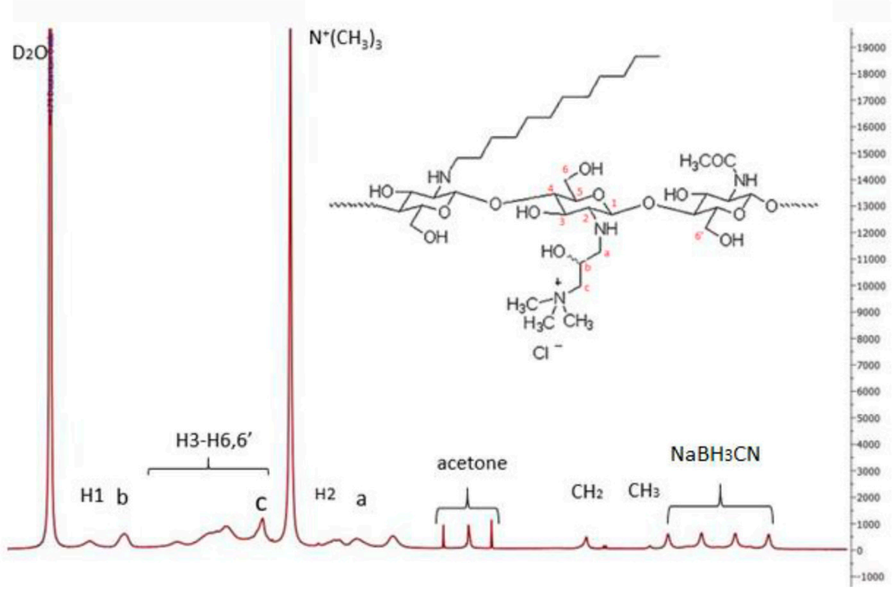

Figure S1. NMR spectrum of the amphiphilic chitosan derivative containing quaternary ammonium and N-dodecyl groups.

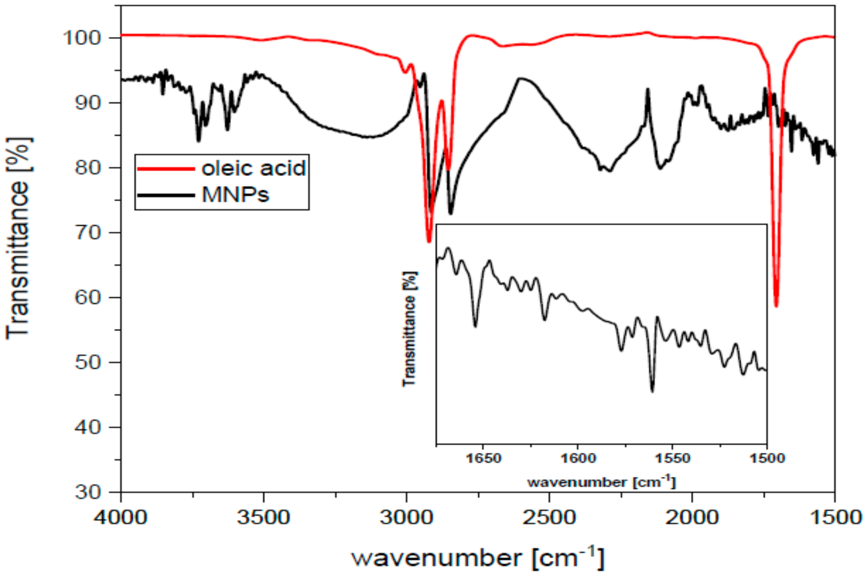

Figure S2. FT-IR spectra of oleic acid and magnetic nanoparticles (MNPs) coated by oleic acid. The inset shows the magnified part of the spectrum.

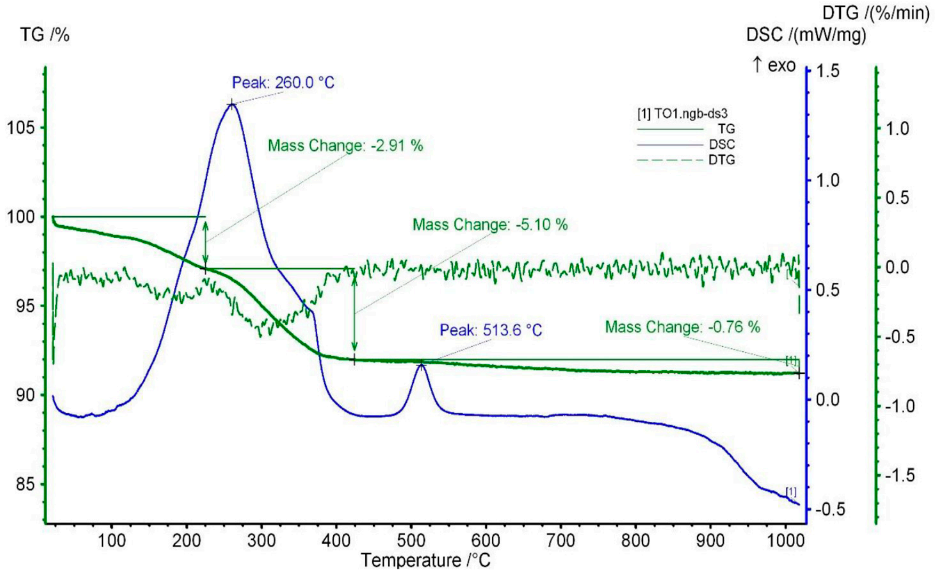

Figure S3. Thermal analysis (TGA-DSC) of the magnetic nanoparticles coated by oleic acid.

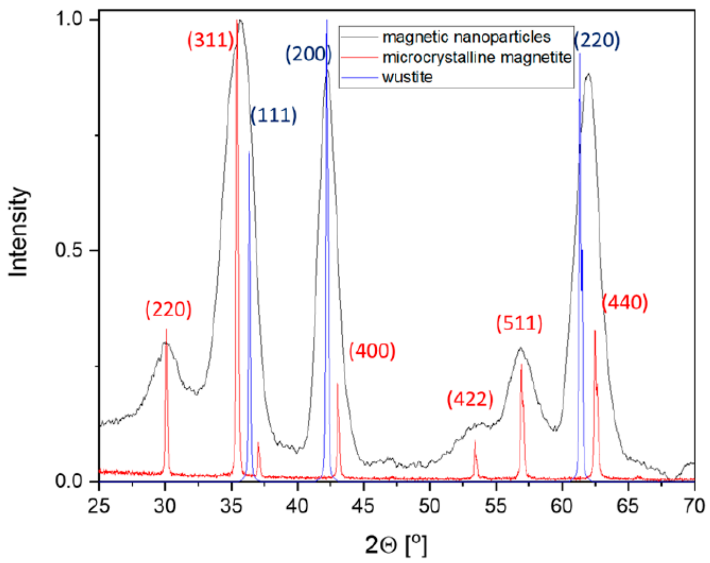

Figure S4. XRD diffraction patterns of magnetic nanoparticles, maghemite and wüstite.

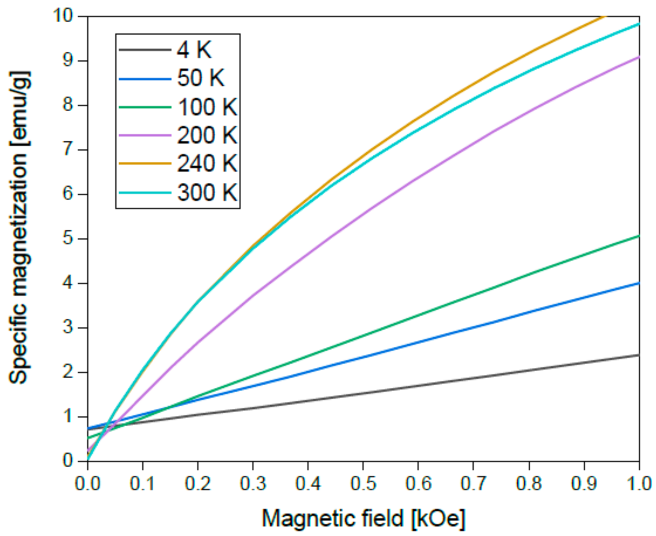

Figure S5. Virgin magnetization curves at 4–300 K temperature range.
